# The C-terminal tail of HIV-1 envelope: a unique role for conserved LLP arginines in Env functional properties

**DOI:** 10.1186/1742-4690-9-S2-P325

**Published:** 2012-09-13

**Authors:** A Kuhlmann, JD Steckbeck, TJ Sturgeon, JK Craigo, RC Montelaro

**Affiliations:** 1 Center for Vaccine Research, Pittsburgh, PA, USA

## Background

The C-terminal tail (CTT) of HIV-1 envelope transmembrane protein has recently been the subject of an increasing number of studies to determine its role in the architecture of the HIV virion and in virus replication. Published studies from our lab have previously demonstrated that the lentivirus lytic peptides (LLP) domains contained in the CTT present highly characteristic and conserved physicochemical and structural properties, including a highly preferential incorporation of arginine over lysine at conserved sites.

## Methods

We previously reported that nonconservative substitution of selected LLP arginines to glutamate residues in a reference provirus resulted in substantial changes in Env structure and function, reflecting an important role of the LLP arginines in overall Env phenotypes. To further evaluate the role of LLP arginines in Env properties, we have now evaluated the effects of conservative substitutions of selected LLP arginines by lysines in the context of the HIV 89.6 provirus. The various lysine substitution mutants are being characterized for Env incorporation, fusogenicity, infectivity, and antigenicity.

## Results

To date, the results of these studies clearly indicate marked changes in Env functional properties as a result of the lysine substitutions for native arginine residues. Thus, these data demonstrate for the first time unique functional properties that are intrinsic to arginine in the LLP domains and that cannot be replaced by the closely related lysine.

## Conclusion

Taken together, these observations reveal the critical role of conserved LLP arginine residues in affecting viral envelope phenotypes and further highlight the role of the CTT as a major determinant of overall HIV Env structural and functional properties. As such, their role as determinants of Env antigenicity, immunogenicity and as conserved epitopes is also investigated in experimental immunizations, and will be informative for future vaccine design.

